# Simultaneously Regulating Uniform Zn^2+^ Flux and Electron Conduction by MOF/rGO Interlayers for High-Performance Zn Anodes

**DOI:** 10.1007/s40820-021-00594-7

**Published:** 2021-02-15

**Authors:** Ziqi Wang, Liubing Dong, Weiyuan Huang, Hao Jia, Qinghe Zhao, Yidi Wang, Bin Fei, Feng Pan

**Affiliations:** 1 Department of Materials Science and Engineering, College of Chemistry and Materials Science, Jinan University, Guangzhou, 510632 People’s Republic of China; 2 School of Advanced Materials, Peking University Shenzhen Graduate School, Shenzhen, 518055 People’s Republic of China; 3 Nano Center, Institute of Textiles and Clothing, Hong Kong Polytechnic University, Hunghom, Kowloon, Hong Kong, 999077 People’s Republic of China

**Keywords:** Zn-based battery, Zn anode, Janus separator, Metal–organic framework, Reduced graphene oxide

## Abstract

Owing to the merits of low cost, high safety and environmental benignity, rechargeable aqueous Zn-based batteries (ZBs) have gained tremendous attention in recent years. Nevertheless, the poor reversibility of Zn anodes that originates from dendrite growth, surface passivation and corrosion, severely hinders the further development of ZBs. To tackle these issues, here we report a Janus separator based on a Zn-ion conductive metal–organic framework (MOF) and reduced graphene oxide (rGO), which is able to regulate uniform Zn^2+^ flux and electron conduction simultaneously during battery operation. Facilitated by the MOF/rGO bifunctional interlayers, the Zn anodes demonstrate stable plating/stripping behavior (over 500 h at 1 mA cm^−2^), high Coulombic efficiency (99.2% at 2 mA cm^−2^ after 100 cycles) and reduced redox barrier. Moreover, it is also found that the Zn corrosion can be effectively retarded through diminishing the potential discrepancy on Zn surface. Such a separator engineering also saliently promotes the overall performance of Zn|MnO_2_ full cells, which deliver nearly 100% capacity retention after 2000 cycles at 4 A g^−1^ and high power density over 10 kW kg^−1^. This work provides a feasible route to the high-performance Zn anodes for ZBs.

**Supplementary Information:**

The online version of this article (10.1007/s40820-021-00594-7).

## Highlights

- Dendrite-free stable Zn plating/stripping was achieved for over 500 h at 2 mA cm^−2^.
- No short-circuit for 10 mAh cm^−2^ of Zn plating.
- Zn|MnO_2_ cells delivered nearly 100% capacity retention over 2000 cycles.

**Supplementary Information:**

The online version of this article (10.1007/s40820-021-00594-7).

## Graphical abstract

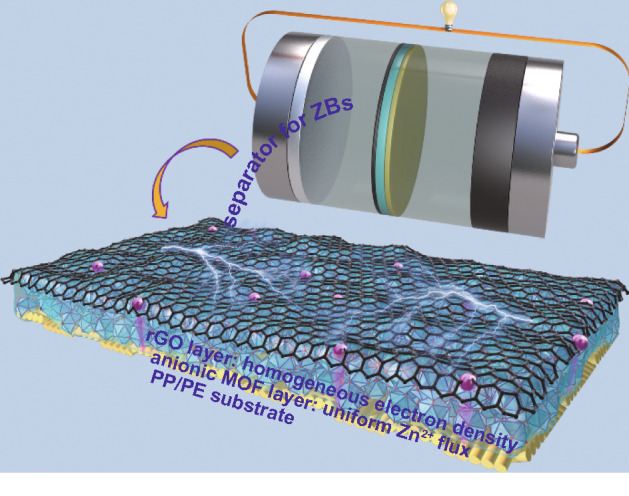

## Introduction

Aqueous Zn-based batteries (ZBs) are considered to be one of the most promising devices for grid-scale energy storage and wearable power sources by virtue of their high safety, environmental benignity, low cost and high energy density [1–6]. Numerous high-performance cathode materials such as manganese oxides [7, 8], cobalt oxides [9], vanadium-based materials [10, 11] and Prussian blue analogs [12] have been strenuously explored en route toward the development of practical ZBs. For the anode, metallic zinc is the best candidate due to its high capacity (820 mAh g^−1^), low redox potential (-0.76 V vs SHE) and easy processing. However, the problems such as dendrite formation, surface passivation and corrosion of Zn anode greatly jeopardize the further development of ZBs [5, 13–15]. To be specific, following a tip-growth mechanism, Zn nucleation usually undergoes at the edges and tips of protuberances on substrate, and a loose and porous layer of harsh dendrites will eventually form after repeated cycling [16]. The inhomogeneous Zn deposition produces “dead Zn” leading to low Coulombic efficiency (CE), and the Zn dendrites bring the risk of separator penetration and battery short-circuit [1]. Another problem of Zn anode is corrosion in aqueous electrolytes, which is resulted from the countless corrosive cells constituted by the potential discrepancy of different regions on the heterogeneous surface of anode [13, 17]. The corrosion process not only continuously consumes electrolyte to generate H_2_ and passivated by-products (ZnO, Zn(OH)_2_ and zincates), but also causes severe self-discharge in ZBs [18].

To circumvent these issues of Zn metal anode, many strategies including structural design, surface coating and electrolyte formulation have been proposed. For instance, 3D porous conductive hosts such as carbon nanotube framework [19], graphene foam [20] and copper foam [21] were exploited as a substrate or supporter for the Zn active material to provide homogeneous nucleation sites and buffer the dendrite growth. For the coating route, Cui and co-workers reported that the lifespan of Zn anode could be extended by 60-fold with a polyamide protecting layer for dendrite-free deposition [22]. In the meanwhile, novel electrolytes, such as “water-in-salt” electrolyte [23] and deep eutectic electrolyte [24], are used to modulate the coordination environment of Zn^2+^ and improve the reversibility of Zn anodes. However, despite the improved electrochemical performance of Zn anodes, the battery energy density was limited by the 3D porous Zn anodes with a low active loading (usually below 10 mg cm^−2^), and the rate capability was restricted by the unconventional electrolytes or coatings with relatively poor Zn^2+^ transport kinetics. Therefore, it is urgently demanded to explore alternative solutions.

Unlike alkali metal (Li, Na and K) anodes in organic electrolytes, no continuous solid electrolyte interphase (SEI) is able to in situ form on the surface of Zn anode in aqueous condition, because H_2_ evolution reaction (HER) usually happens before the decomposition of routine anions or organic solvents [25, 26]. The insulated byproducts intermittently generated by HER result in an uneven charge distribution on Zn anode [27]. The deposition on metallic anodes is controlled by the transport of both electrons and ions [28]. Therefore, besides uniform Zn^2+^ flux, homogeneous electron distribution should be taken into consideration as well to design a high-performance Zn anode. To this end, we report here a Janus separator based on anionic metal–organic framework (MOF) and reduced graphene oxide (rGO) bifunctional interlayers. The anionic MOF provides a Zn^2+^-rich layer near the surface of Zn anode and also regulates uniform Zn^2+^ flux with the inner well-defined anionic sub-nano tunnels. Meanwhile, the rGO serves as a stable electro-conductive layer on top surface of Zn anode, which not only reduces Zn^2+^/Zn redox barrier on current collector but also increases the reversibility of Zn anode by digesting “dead Zn” during cycling. Moreover, through diminishing the microscopic potential discrepancy on Zn surface, the rGO layer effectively mitigates the corrosion of Zn anodes during battery storage. Benefited from the Janus separator with MOF/rGO functional interlayers, the CE, lifespan and rate capability of Zn anode are significantly improved, and a high-performance Zn|MnO_2_ battery is obtained.

## Experimental Section

### Materials

#### MOF Material

0.22 g 1,3,5-Benzenetricarboxylic acid and 0.34 g ZrOCl_2_·8H_2_O were dissolved in 40 mL mixed solvent of N,N-dimethylformamide and formic acid (v: v = 1: 1). Then, the solution was transferred into a 100-mL Teflon-lined autoclave and heated at 100 °C for 2 days. The MOF was then collected by filtration, washed with ethanol and dried in vacuum at 60 °C. After that, the MOF was dispersed in 40 mL 1 M HCl and stirred at 90 °C for 12 h to prepare the anionic MOF. Zn^2+^ was introduced in the MOF by cationic exchange. Typically, 0.5 g anionic MOF was socked in 7 mL 1 M zinc acetate solution under stirring for 5 days. The zinc acetate solution was refreshed every 24 h. The synthesis procedure can also be found elsewhere [29]. To determine the ionic conductivity, the MOF powder along with several drops of deionized water was pressed into a pellet with a stainless-steel die. Then, the pellet was sandwiched between two stainless-steel electrodes to perform the EIS tests. The ionic conductivity (*σ*) was calculated by the equation, *σ* = *L/RA*, where *L* and *A* are the thickness and area of the pellet, and *R* is interfacial resistance.

#### Janus Separator

0.27 g MOF was dispersed in 1.5 mL N,N-dimethylformamide (DMF) and 8 mL acetone through ultrasonic vibration, to which 0.4 g 7.5 wt% poly(vinylidene fluoride) (PVDF) DMF solution was added. With continuous stirring at 105 °C, acetone in the mixture was evaporated gradually and the remaining slurry was coated onto a piece of PP/PE hydrophilic non-woven separator (Mitsubishi Paper Mills Ltd.) with a doctor blade. After dried at 80 °C in vacuum, the MOF-coated separator was cut into ϕ 19 mm round pieces. 3 mL aqueous dispersion containing 0.15 mg rGO (XF019, XFNANO) and 0.15 mg polytetrafluoroethylene (PTFE, used as binder) was then filtered directly onto the MOF-coated separator. The Janus separator was dried at room temperature. The MOF and rGO loadings are about 1.8 and 0.075 mg cm^−2^, respectively. For the half-Janus separator, half of the MOF-coated separator was masked with a piece of tape. After filtration with 1.5 mL rGO/PTFE dispersion, the tape was peeled off along with the MOF coating on the half side.

#### β-MnO_2_@rGO

Typically, 0.5 g MnSO_4_·H_2_O and 2 mL 0.5 M H_2_SO_4_ were dissolved in 60 mL H_2_O, to which 4 mL GO aqueous dispersion (XF020, XFNANO) was added. Under continuously stirring, 20 mL 0.1 M KMnO_4_ was dropwise added to the solution. Afterward, the mixture was stirred and ultrasonicated at room temperature for another 30 min before it was sealed in a 100-mL autoclave and heated at 180 °C for 12 h. *β*-MnO_2_@rGO was collected by filtration, rinsed with H_2_O and dried at 60 °C in vacuum.

#### Battery Assembly

CR2032 coin cells were assembled for battery tests. The cathode containing *β*-MnO_2_@rGO, acetylene black (Super P) and PVDF in a weight ratio of 7: 2: 1 was used to assemble the Zn|MnO_2_ batteries. The MnO_2_ loading is 1.5–2.0 mg cm^−2^. Aqueous solution of 2 M ZnSO_4_ and 0.1 M MnSO_4_ was used as electrolyte (50 μL mg^−1^) and a Zn plate was used as anode. The functionalized side of Janus separator faced toward Zn anode. For the assembly of Zn|SS, Zn|Cu and Zn|Zn cells, 2 M ZnSO_4_ aqueous solution was used as electrolyte. The functionalized side of Janus separator faced toward SS electrode and Cu current collector in Zn|SS and Zn|Cu cells, respectively, and two pieces of separator were used in Zn|Zn symmetric cells with functionalized side facing toward both Zn electrodes.

### Characterizations

XRD patterns were recorded by a Bruker D8 Advance diffractometer using Cu Kα, *λ* = 1.541 Å. SEM images and EDS mappings were taken with a Zeiss Supra 55 scanning electron microscope. TEM study of *β*-MnO_2_@rGO was performed on a JEOL JEM-2100F instrument. EIS, CV and LSV measurements were conducted with a Solartron Analytical electrochemical workstation. Cycling performance of Zn|Cu, Zn|Zn and Zn|MnO_2_ cells was tested by a LAND CT2001A battery test system. The sheet resistance of the Janus separator was tested using a KDY-1 four-probe square resistance tester.

## Results and Discussion

**Fig. 1:**
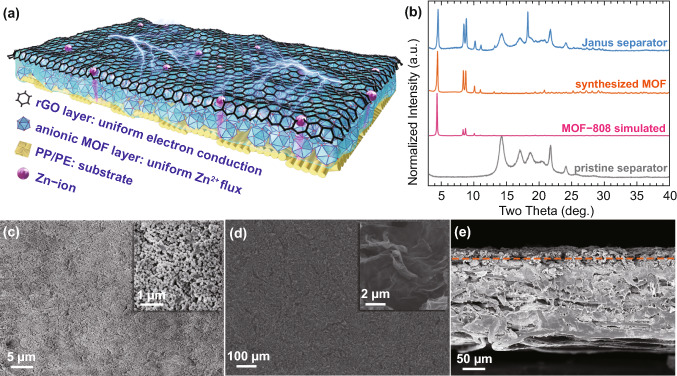
**a** Schematic illustration for the Janus separator. **b** XRD patterns of the simulated MOF-808, synthesized MOF, pristine separator and Janus separator. SEM images of the **c** MOF and **d** rGO layer and the **e** cross-section view of the Janus separator (Dash line: the boundary between substrate and MOF/rGO interlayers)

The structure of the Janus separator (Fig. S1) with laminated ionic/electro- conductive layers is illustrated in Fig. 1a. An electrochemically stable Zr-based MOF serves as the ionic conductive layer, which is constructed by anionic MOF-808 [30, 31] frameworks and incorporated Zn^2+^ counter ions. The ionic conductivity of MOF is tested to be 3 × 10^–4^ S cm^−1^ at room temperature. The synthesized MOF demonstrates identical X-ray diffraction (XRD) pattern with the reported MOF-808 (Fig. 1b), confirming its high phase purity. Meanwhile, its structural integrity after being coated on a piece of polypropylene/polyethylene (PP/PE) substrate, which is used as the pristine separator for reference as well, is also validated by the strong MOF reflection peaks of the fabricated Janus separator. The scanning electron microscope (SEM) image of the MOF coating in Fig. 1c shows that spherical MOF crystals with a diameter of about 100 nm are tightly arranged into a dense and uniform layer on the PP/PE substrate. Through filtration, the rGO nanosheets homogeneously deposit on top of MOF layer forming a smooth surface morphology (Fig. 1d). Besides high ionic conductivity, good electro-conductivity is also essential for the functional interlayers to favor uniform Zn deposition. Small sheet resistance of 474 Ω sq^−1^ was observed for the Janus separator owing to the good conductivity of the continuous rGO layer. The SEM image and corresponding energy-dispersive X-ray spectroscopy (EDS) elemental mappings for the cross-section view of Janus separator are displayed in Figs. 1e and S2, and the total thickness of the MOF/rGO layers is measured to be 25 μm accordingly. The electrochemical window of the Janus separator in 2 M ZnSO_4_ aqueous electrolyte was tested with a Zn|stainless-steel (SS) asymmetric cell, as shown in Fig. S3. Evidently, due to the oxygen evolution reaction (OER) of water splitting [26], the high potential limit for the Zn|SS cell with pristine and Janus separator is restricted to about 2.3 V. While in the cyclic voltammetry (CV) scan at low potential range, both plating and stripping overpotentials (-0.075 and 0.17 V, respectively) of Zn are decreased with Janus separator, indicating a lower Zn^2+^/Zn redox energy barrier benefitted from the functional MOF/rGO interlayers. The Zn^2+^ transference number (t_Zn_^2+^) with the two separators was tested by the Evans’ method [32] using Zn|Zn symmetric cells. As shown in Fig. S4, the t_Zn_^2+^ of pristine separator in ZnSO_4_ aqueous electrolyte is 0.29, similar to the reported values [26, 33]. In sharp contrast, the Janus separator shows an increased t_Zn_^2+^ of 0.55, promising for a more effective Zn^2+^ conduction. The significantly increased t_Zn_^2+^ can be attributed to the anionic MOF layer, which not only provides additional conductive Zn^2+^ cations, but also electrostatically repulses the transport of SO_4_^2−^ anions [34].

**Fig. 2:**
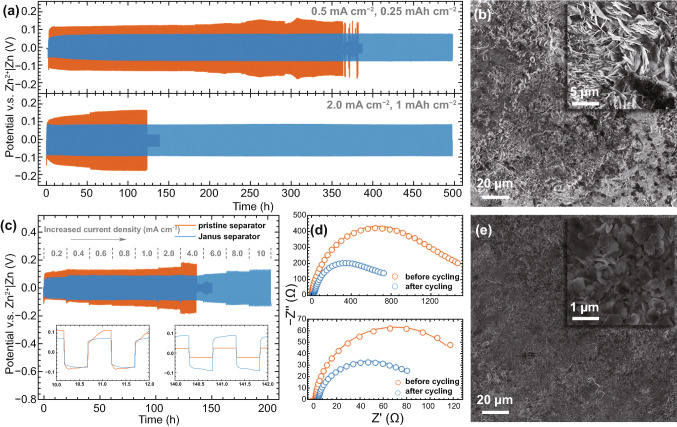
**a** Long-term cycling performance of the Zn|Zn symmetric cells with pristine (orange line) and Janus (blue line) separator at 0.5 and 2.0 mA cm^−2^. SEM images of the Zn anode foils after cycling at 2 mA cm^−2^ for 100 cycles with **b** pristine and **e** Janus separator. **c** Cycling performance of the Zn|Zn symmetric cells with pristine and Janus separator at different current densities. Inset: Zn plating/stripping profiles for the selected cycles. **d** EIS for the Zn|Zn symmetric cells with pristine (top) and Janus (bottom) separator before and after cycling at 2 mA cm^−2^ for 100 cycles from 10^6^ to 0.1 Hz. Circles: experimental; solid lines: simulated

The superior performance of Janus separator for supporting Zn anode was demonstrated under galvanostatic conditions with Zn|Zn symmetric cells. The MOF/rGO on the separator serves as interlayers between electrolyte and Zn anode to simultaneously regulate Zn^2+^ flux and electron conduction for uniform Zn deposition. As depicted in Fig. 2a, with the Janus separator, the cell demonstrates stable long-term cycling over 500 h without any voltage fluctuation or short-circuit, and the overpotential is only about 75 and 89 mV under current densities of 0.5 and 2.0 mA cm^−2^, respectively. The cell with pristine separator showed unstable voltage profiles with much larger overpotential and became short-circuit after 360 and 120 h cycling at 0.5 and 2.0 mA cm^−2^, respectively, owing to the Zn dendrites formed during repeated Zn plating/stripping. Furthermore, the cycling performance of the Zn|Zn symmetric cells was examined under different current densities. As shown in Fig. 2c, even at a super high current density of 10 mA cm^−2^, the cell with Janus separator still demonstrates a stable cycling behavior. However, in stark contrast, the cell with pristine separator became short-circuit when the current density increased to 4 mA cm^−2^. Moreover, with Janus separator, the voltage plateau in each plating/stripping cycle is flatter and smoother compared with the counterpart, as illustrated in the insets of Fig. 2c, indicating a stable interface for Zn deposition. To elucidate such superior performance, the interfacial resistance and Zn anode morphologies of the Zn|Zn cells after cycling (after the 100^th^ cycle of the test in Fig. 2c) were also investigated. As shown by the electrochemical impedance spectroscopy (EIS) profiles in Fig. 2d, the freshly assembled cell with pristine separator shows a high charge transfer resistance (*R*_ct_) of 1034 Ω, indicating a poor interface for Zn plating/stripping. With the Janus separator, however, low *R*_ct_ of 138 Ω is observed, almost one-eighth of its counterpart, demonstrating good compatibility between MOF/rGO interlayers and Zn anodes. After cycling, the *R*_ct_ decreased to about 70 Ω due to the interfacial reconstruction by repeated Zn plating/stripping, and it is still smaller than the value (498 Ω) with pristine separator. The small *R*_ct_ is well consistent with the low overpotential of the voltage profiles in Fig. 2a, c. We found that the rGO layer partially transferred on the surface of Zn anode after cycling (Fig. S5), indicating a seamlessly contact between them. Subsequently, the SEM morphologies of Zn anodes after cycling are shown in Fig. 2b, e. With pristine separator, a porous and rough surface covered by sharp Zn flakes is observed, which is the main reason for the battery short-circuit. In the scenario of Janus separator, the Zn anode demonstrates a glabrous and dense surface owing to the uniform Zn^2+^ flux and electron conduction regulated by the MOF/rGO interlayers. At higher magnification (inset of Fig. 2e), many micro-sized Zn hexagonal crystals parallel to the anode surface were distinguished. Notably, recent research found that the rGO with a small lattice mismatch for Zn metal can induce planar nucleation and epitaxial growth of Zn [35, 36]. In our case, the confined crystallographic orientation of Zn deposition is also regulated by the Janus separator to avoid dendrite growth. To elucidate the synergistic effect of MOF and rGO on the performane of Zn anode, separators with only MOF or rGO layer are prepared and tested. As shown in Fig. S6, they suffer from large overpotential and short cycling life, respectively, as compared with the voltage profile of Janus separator. Such result indicates the promoted Zn anode is attributed to both homogeneous ion flux and electron conduction.

**Fig. 3:**
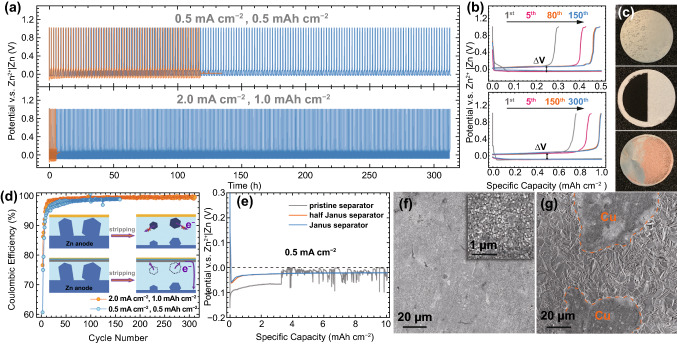
**a** Cycling performance of the Zn|Cu cells with pristine (orange line) and Janus (blue line) separator at 0.5 and 2.0 mA cm^−2^. **b** Voltage profiles of the Zn|Cu cell with Janus separator for the selected cycles at 0.5 (top) and 2.0 (bottom) mA cm^−2^. **d** CE of the Zn|Cu cells with a Janus separator. Inset: the proposed mechanism for high CE showing the Zn anodes with pristine (up) and Janus (bottom) separator, where the dark blue hexahedrons represent “dead Zn.” **e** Zn plating profiles of Zn|Cu cells with different separators at 0.5 mA cm^−2^. **c** Photographs of the Zn anode (top) and Cu current collector (bottom) after Zn plating with 10 mAh cm^−2^ with a half-Janus separator (middle). SEM images for the Cu current collector after Zn plating on the **f** functionalized and **g** pristine side of a half-Janus separator

The positive effect of Janus separator on promoting the performance of Zn anode was further evaluated in a Zn|Cu cell configuration. Figure 3a shows the voltage profiles of Zn plating/stripping on Cu current collector at 0.5 and 2.0 mA cm^−2^. Without functional MOF/rGO interlayers, the cell got short-circuit easily, especially under a large current density. We disassembled the failed cell and confirmed that the short-circuit was caused by the growth of Zn dendrites, as observed on the separator of the failed cell (Fig. S7). Facilitated by the Janus separator, the cycling life of the Zn|Cu cell could be prolonged over 310 h. As commonly known, the low Coulombic efficiency of Zn anode is attributed to the generation of passivation by-products (ZnO, etc.) and anode pulverization, which may cut off electronic percolation and cause “dead Zn” [23, 37]. Fortunately, owing to the conductive rGO layer on top of deposited Zn to provide additional electronic pathways, the “dead Zn” can be digested during cycling. After 100 cycles, the cell demonstrates elevated CE (Fig. 3d) of about 97.8% and 99.2% at 0.5 and 2.0 mA cm^−2^, respectively. Such mechanism for the high CE is schematically illustrated in the inset of Fig. 3d. Figures 3b and S8 show the Zn plating/stripping voltage profiles of Zn|Cu cells for the selected cycles. After introducing the functional MOF/rGO interlayers, the Zn nucleation overpotential on Cu current collector, which can be defined by the difference between the sharp tip and the later plateau of the deposition voltage profile [38], is significantly reduced. Moreover, the voltage hysteresis (ΔV) also decreased from 0.20 to 0.10 V at 0.5 mA cm^−2^, and from 0.26 to 0.14 V at 2.0 mA cm^−2^, demonstrating the Zn plating/stripping kinetics was promoted. To make better comparison between the two separators, a half-functionalized separator (Fig. 3c, middle) was prepared. As displayed in Fig. 3e, even under a large Zn deposition amount of 10 mAh cm^−2^, the voltage profiles of the Zn|Cu cells with Janus and half-Janus separator are smooth and steady, showing superior performance than the cell with pristine separator, which short-circuited after 3.2 mAh cm^−2^ deposition. As observed in the Zn anode (Fig. 3c, top) and Cu current collector (Fig. 3c, bottom) after running for 5 plating/stripping cycles, the Zn deposition mainly happened on the functionalized side and resulted in a uniform and dense deposition layer, as shown by the SEM in Fig. 3f. In sharp contrast, only aggregations of large Zn flakes could be observed on the blank side of Cu current collector (Fig. 3g). These results conclusively confirm that the functional MOF/rGO interlayers effectively facilitate the performance of Zn anode through guiding homogeneous Zn deposition and reducing Zn^2+^/Zn redox barrier.

**Fig. 4:**
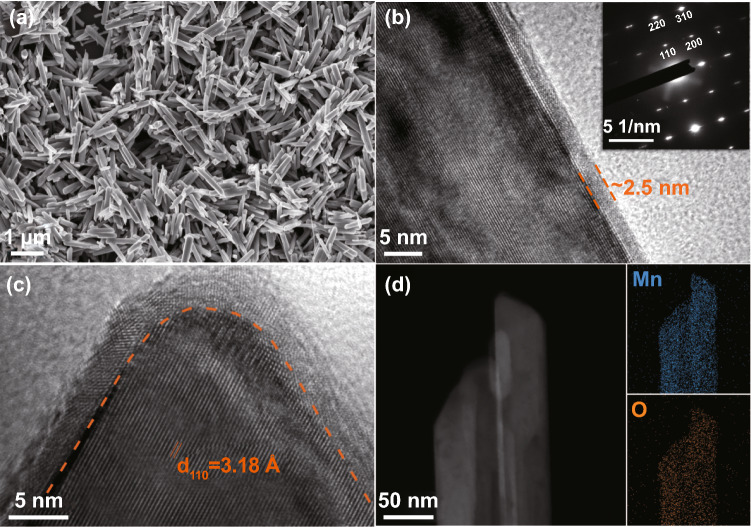
**a** SEM and **b**, **c** HRTEM images of the synthesized *β*-MnO_2_@rGO. **d** STEM image and corresponding elemental mappings of *β*-MnO_2_@rGO

**Fig. 5:**
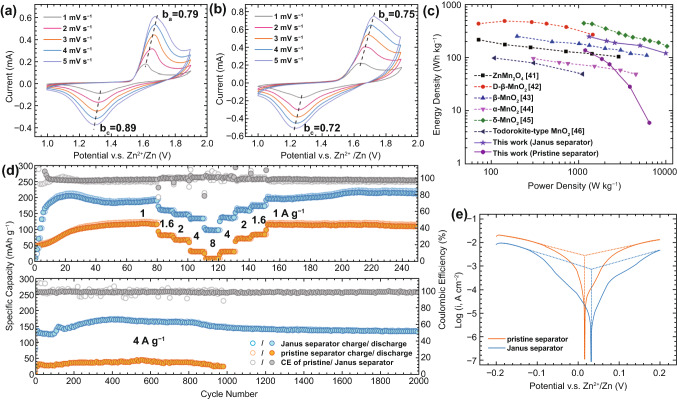
CV profiles of the Zn|MnO_2_ batteries with **a** Janus and **b** pristine separator. **c** Ragone plots of the reported Zn|MnO_2_ battery systems. **d** Rate capability (top) and long-term cycling performance (bottom) of the Zn|MnO_2_ batteries with pristine and Janus separator. **e** Linear polarization curves of the Zn|Zn symmetric cells with Janus and pristine separators at a scan speed of 1 mV s^−1^

All these superiorities were further investigated in aqueous Zn|MnO_2_ full batteries with a cell configuration schemed in Fig. S9. The cathode material, *β*-MnO_2_@rGO nanorods (Fig. 4a), was synthesized through a hydrothermal reaction. As confirmed by the high-resolution transmission electron microscope (HRTEM) images in Fig. 4b, c, a thin layer (about 2.5 nm) of rGO was successfully in situ coated on the nanorods for better electronic conductivity. The elemental mapping images in Fig. 4d show that the Mn and O elements are evenly distributed in *β*-MnO_2_@rGO nanorods. The phase purity and good crystallinity of the synthesized MnO_2_ cathode material were identified by its sharp XRD pattern in Fig. S10. In the CV profiles of the Zn|MnO_2_ cells (Fig. 5a, b), both a low potential shift of the anodic peak and a high potential shift of the cathodic peak are observed after introducing the functional MOF/rGO interlayers, implying that the Janus separator facilitates faster charge/discharge kinetics. Such result is consistent with the tests of Zn|Zn and Zn|Cu cells. Moreover, the CV measurement can be used to distinguish different charge storage processes, as the peak current (*i*) and scan rate (*v*) obey the power law [39, 40]:

$$i = av^{b}$$

where the parameter *b* denotes the charge storage process. A *b* value of 0.5 indicates a typical diffusion-dominated process that of 1.0 demonstrates a capacitive process. The *b* values for the anodic/cathodic peaks of the cells using pristine and Janus separator are calculated to be 0.75/0.72 and 0.79/0.89, respectively (Fig. S11). The increased *b* values suggest that the functional MOF/rGO interlayers favor the capacitive process and enable elevated charge storage speed of the Zn|MnO_2_ battery. Benefited from such merits, the electrochemical performance of Zn|MnO_2_ battery is saliently improved. As shown in Fig. 5d, with Janus separator, the capacities under different current rates are much higher than these of the cell with a pristine separator. Particularly, a reversible capacity of 97 mAh g^−1^ was achieved at 8 A g^−1^, which is more than 13 times higher than its counterpart. The remaining capacity (210 mAh g^−1^) at 1 A g^−1^ after 250 cycles was also doubled compared with that (108 mAh g^−1^) of pristine separator. Moreover, the long-term cycling stability of Zn|MnO_2_ batteries was investigated at 4 A g^−1^. The cell demonstrated stable cycling (nearly 100% capacity retention) with a reversible capacity of 135 mAh g^−1^ after 2000 cycles, far beyond that of the counterpart (25 mAh g^−1^ after 1000 cycles). We further compared the EIS of the Zn|MnO_2_ batteries before and after cycling, as displayed in Fig. S12. The battery using a Janus separator shows much smaller resistance. The significantly reduced semicircle in high frequency region of the EIS, which corresponds to the ion migration in the passivation layer of Zn anode, indicates a stabilized Zn anode. As facilitated by the Janus separator, the power densities (about 10 kW kg^−1^) rank the top level among reported Zn|MnO_2_ batteries [41–46], as summarized in the Ragone plot (Fig. 5c). Despite the debatable reaction mechanism, it is commonly accepted that the capacity of β-MnO_2_ in mildly acidic electrolyte is mainly contributed by reversible proton insertion/desertion [42, 47, 48]. The diffusion of proton is fast due to its small size, and thus, the battery kinetics is controlled by the redox of Zn^2+^/Zn at anode, especially under high current densities. Therefore, by facilitating a high-performance Zn anode, the Janus separator dramatically promotes the overall performance of Zn|MnO_2_ battery. Corrosion of Zn anode is another notorious problem of aqueous ZBs, which causes H_2_ evolution and Zn passivation. The corrosive cells constituted by the microscopic potential discrepancy on Zn anode surface exacerbate such a process [49]. Fortunately, the conductive rGO layer on top of Zn anode is able to short-circuit the corrosive cells, diminish the microscopic potential discrepancy and relieve the corrosion problem. To examine the anode passivation caused by Zn corrosion, XRD studies were performed on the Zn anodes of the freshly assembled Zn|MnO_2_ batteries after dwelling for 24 h. As shown in Fig. S13, reflection peaks of zinc hydroxide sulfate (Zn_4_SO_4_(OH)_6_·4H_2_O) are observed for the anode with pristine separator, but no byproduct can be detected for the scenario of Janus separator, indicating a slow corrosion rate. The corrosion of Zn anode was further characterized by the linear polarization tests of Zn|Zn symmetric cells. As depicted in Fig. 5e, the anode exhibits both increased corrosion potential (from 0.016 to 0.032 V) and decreased corrosion current (from 2.63 to 0.71 mA cm^−2^) under the protection of MOF/rGO interlayers, elucidating a higher electrochemical stability against corrosion in electrolyte [50]. Finally, the stability of the MOF layer on Janus separator after battery cycling was validated by the strong characteristic XRD peaks of MOF (Fig. S14).

## Conclusions

In summary, the performance of Zn anode in mild aqueous electrolytes has been promoted by a Janus separator with MOF/rGO bifunctional interlayers. Owing to the uniform Zn^2+^ flux and electron conduction regulated by the anionic MOF and rGO, a stable cycling of Zn|Zn symmetric cells was achieved over 500 h, and the short-circuit was effectively prohibited even under an elevated current density of 10 mA cm^−2^. Meanwhile, the Zn|Cu asymmetric cells also demonstrated high Coulombic efficiency of 99.2% after 100 cycles, indicating increased reversibility of Zn anodes. Moreover, according to the plating/stripping voltage profiles and CV tests, the Janus separator reduced the Zn nucleation and deposition barrier on the current collector and thus promoted the Zn redox kinetics. We also found that the corrosion of Zn anodes was alleviated using the Janus separator, because the microscopic potential discrepancy on Zn anode was diminished by the rGO layer with homogeneous electron conduction. Benefited from all these merits of the functional MOF/rGO interlayers, the Zn|MnO_2_ battery exhibited good long-term cycling stability over 2000 cycles and high-rate capability up to 8 A g^−1^, far beyond the reference cell using a pristine separator. Such a successful paradigm, simultaneously regulating uniform ion flux and electron conduction, can be envisaged for other battery systems based on metallic anodes.

## Supplementary Information

Below is the link to the Supplementary Information.

Supplementary file 1 (PDF 1053 kb)
