# Supplementary material for: Simultaneously Regulating Uniform Zn2+ Flux and Electron Conduction by MOF/rGO Interlayers for High-Performance Zn Anodes
Source: Nanomicro Lett. 2021 Feb 15;13:73. doi: 10.1007/s40820-021-00594-7 (PMC8187534; doi:10.1007/s40820-021-00594-7)
Supplement: Supplementary file 1 — (PDF 1053 kb) [file 40820_2021_594_MOESM1_ESM.pdf]

Supporting Information for

Simultaneously Regulating Uniform Zn^{2+} Flux and Electron Conduction by MOF/rGO Interlayers for High-Performance Zn Anodes

Ziqi Wang[1,#], Liubing Dong[1,#] Weiyuan Huang[2], Hao Jia[3], Qinghe Zhao[2], Yidi Wang[3], Bin Fei[3,*], Feng Pan[2,*]

[1]Department of Materials Science and Engineering, College of Chemistry and Materials Science, Jinan University, Guangzhou 510632, P. R. China

[2]School of Advanced Materials, Peking University Shenzhen Graduate School, Shenzhen 518055, P. R. China

[3]Nano Center, Institute of Textiles and Clothing, Hong Kong Polytechnic University, Hunghom, Kowloon, Hong Kong 999077, P. R. China

[#]Ziqi Wang and Liubing Dong contributed equally to this work

*Corresponding authors. E-mail: panfeng@pkusz.edu.cn (Feng Pan), bin.fei@polyu.edu.hk (Bin Fei)

Supplementary Figures

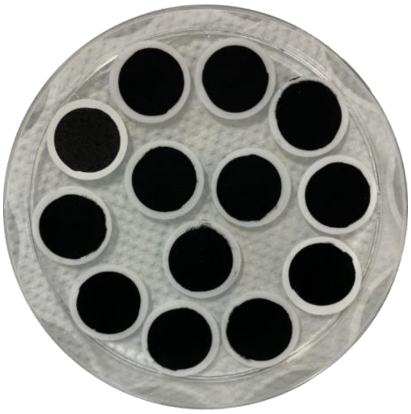

Fig. S1 Photograph of the as prepared Janus separators

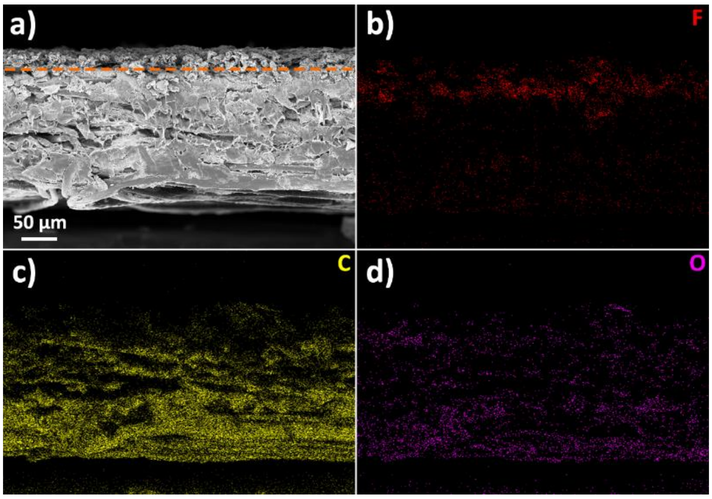

Fig. S2 a SEM image and **b-d** corresponding EDS elemental mappings for the cross-section view of the Janus separator

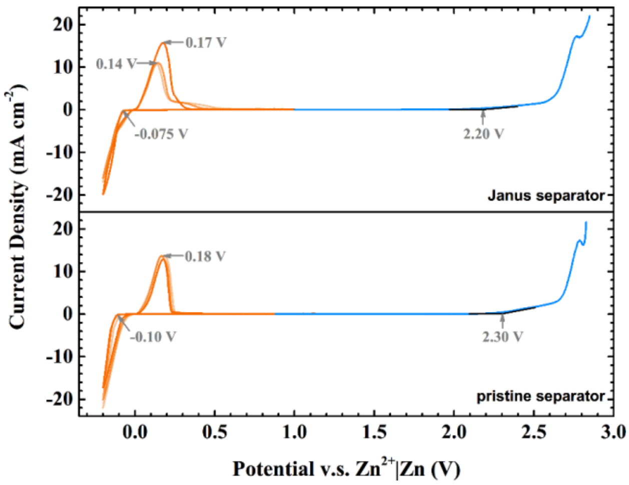

Fig. S3 LSV (blue line) and CV (deep to light orange lines represent the 1st to 3rd cycles) profiles for the electrochemical window test with pristine and Janus separator in 2 M $ZnSO_4$ aqueous electrolyte. The scan rate is 0.2 mV s^{-1}

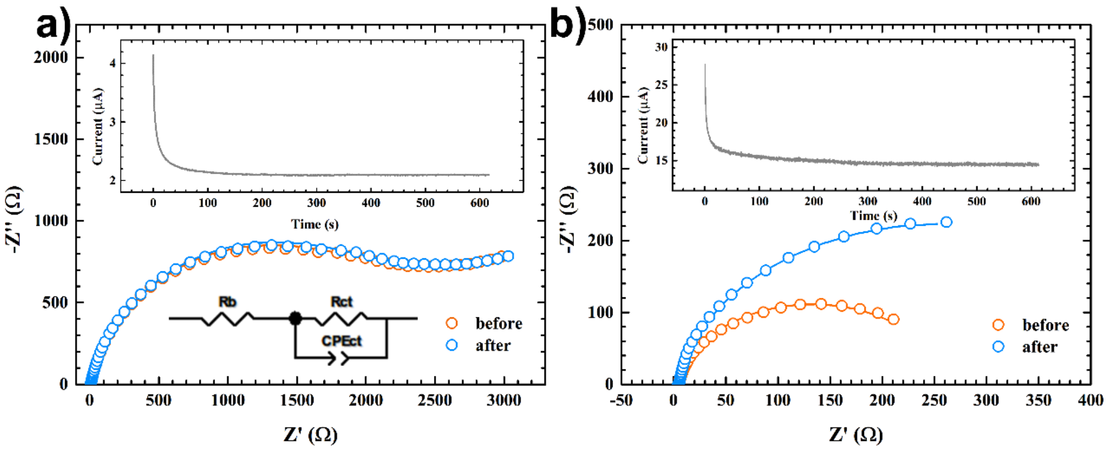

Fig. S4 Zn^{2+} transference number tests for **a** pristine and **b** Janus separator in 2 M $ZnSO_4$ electrolyte

The Zn^{2+} transference number was calculated based on the equation $t_{Zn^{2+}} = \frac{I_s(\Delta V - I_0 R_o)}{I_0(\Delta V - I_s R_s)}$, where I_0/ I_s and R_0/ R_s are the initial/ steady current and interfacial resistance, respectively. ΔV is set as 10 mV. The I_0/ I_s, and R_0/ R_s for pristine and Janus separator are 4.15/2.09 μA, 1477/1570 Ω and 27.7/14.4 μA, 256.2/505 Ω, respectively.

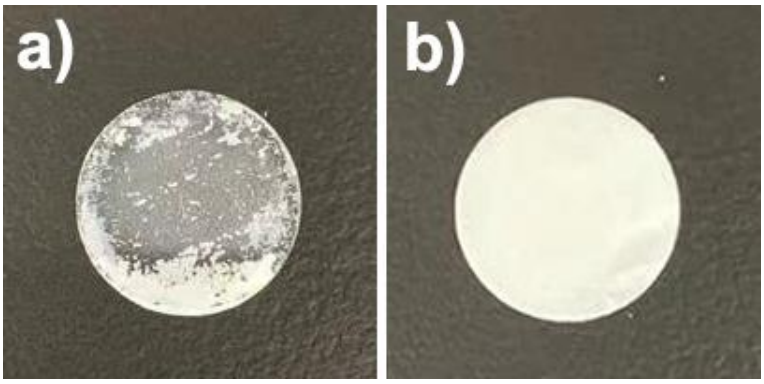

Fig. S5 Photographs of the Zn anode after cycling: **a** upon disassembly and **b** after ultrasonic cleaning

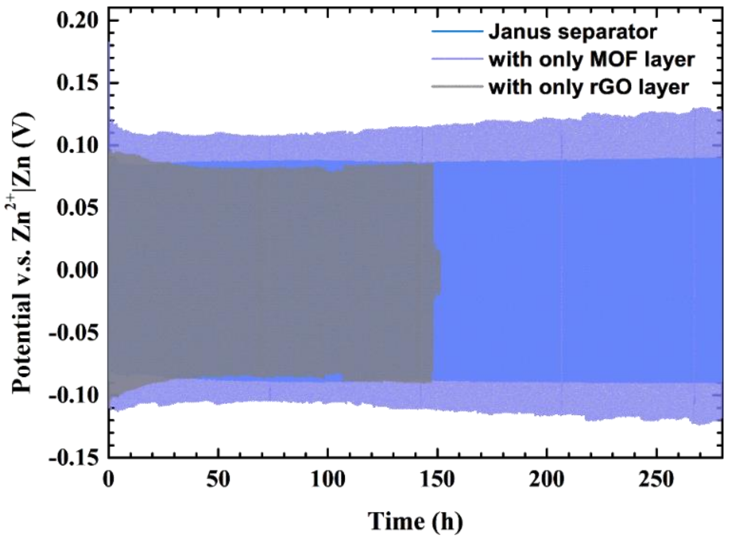

Fig. S6 Cycling performance of the Zn|Zn symmetric cells using the separator with only MOF or rGO layer at 2.0 mA cm^{-2}, 1 mAh cm^{-2}

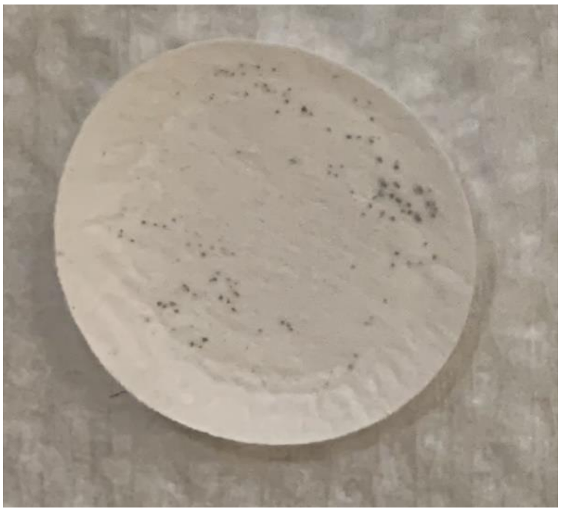

Fig. S7 Photograph of the pristine separator of Zn|Cu cell after short-circuit

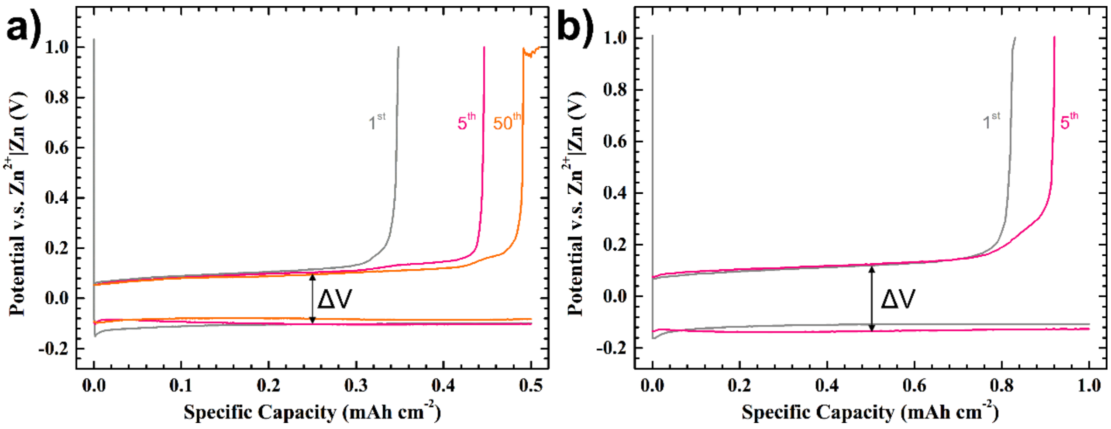

Fig. S8 Voltage profiles of the Zn|Cu cell with pristine separator for the selected cycles at **a** 0.5 and **b** 2.0 mA cm^{-2}

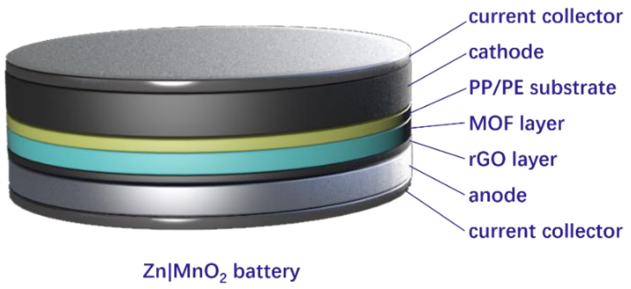

Fig. S9 Illustration of Zn|MnO_2 battery configuration

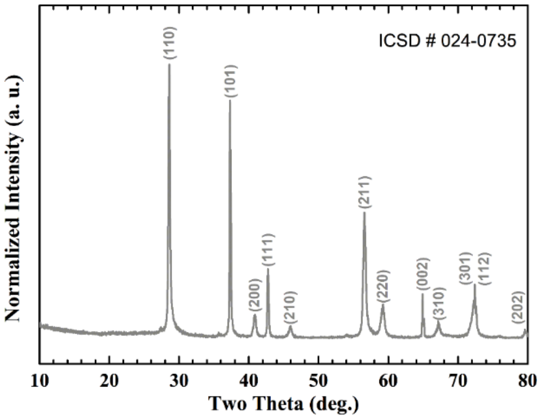

Fig. S10 XRD pattern of the synthesized β-MnO_2@rGO

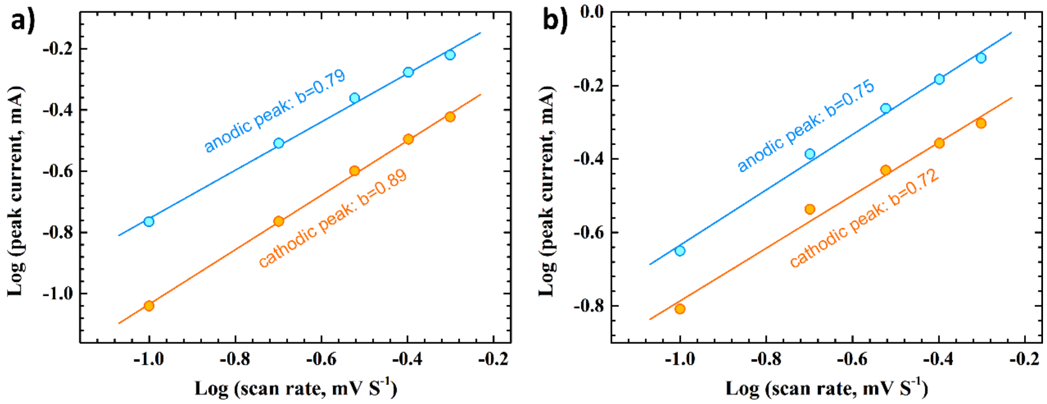

Fig. S11 Calculation of *b* values for the cells with **a** Janus and **b** pristine separators

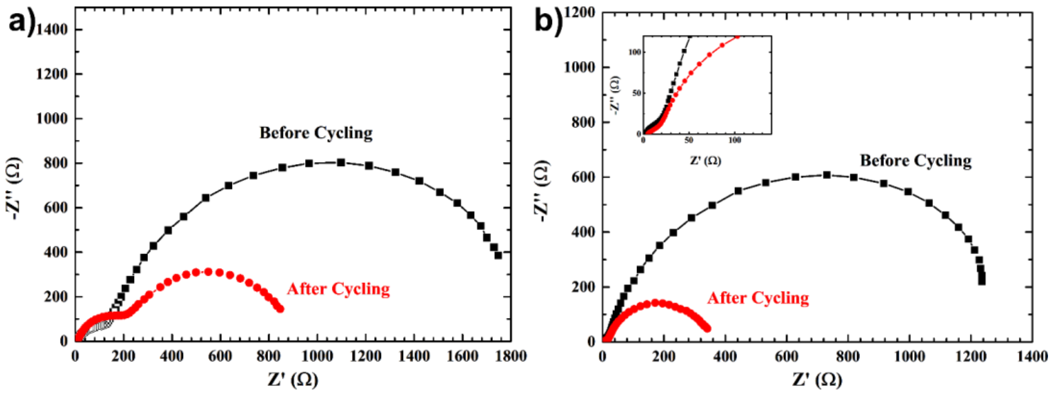

Fig. S12 EIS profiles of the batteries with **a** pristine and **b** Janus separators before cycling and after 50 cycles at 4 A g^{-1} charge/discharge rate

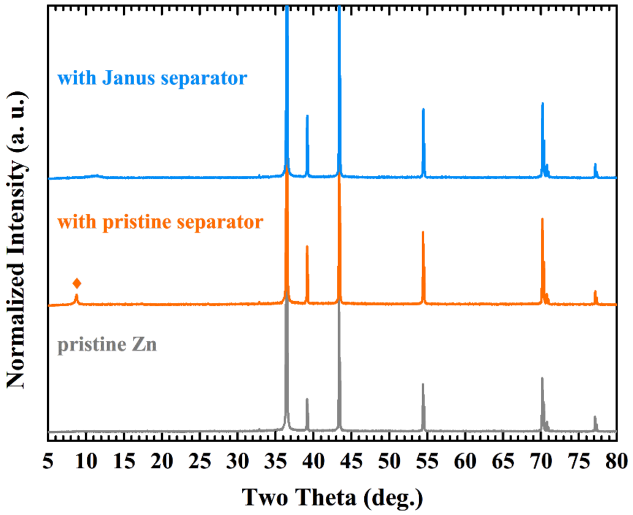

Fig. S13 XRD for the Zn anodes of the disassembled cells. The refection peak of zinc hydroxide sulphate ($Zn_4SO_4(OH)_6·4H_2O$, PDF No. 44-0673) is marked with a diamond

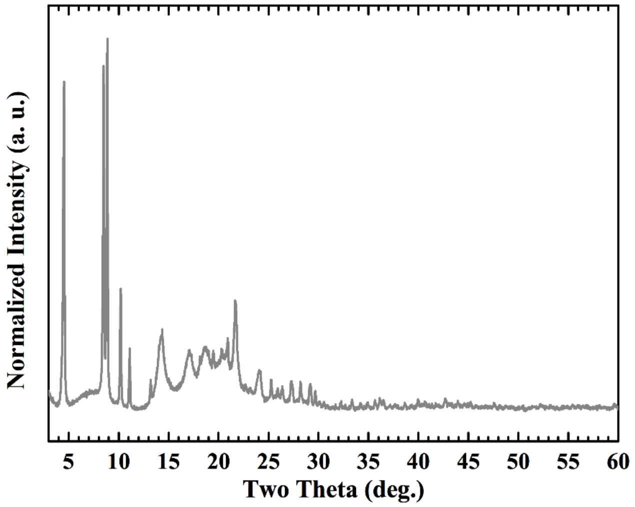

Fig. S14 XRD of the Janus separator after battery cycling
